# Fertility-sparing management in cervical cancer: balancing oncologic outcomes with reproductive success

**DOI:** 10.1186/s40661-016-0030-9

**Published:** 2016-10-21

**Authors:** Karla Willows, Genevieve Lennox, Allan Covens

**Affiliations:** 1 Division of Gynecologic Oncology, Department of Obstetrics and Gynecology, University of Toronto, M700-610 University Avenue, Toronto, M5G 2 M9 ON Canada; 2 Division of Gynecologic Oncology, T2051 Odette Cancer Centre, University of Toronto, 2075 Bayview Avenue, Toronto, M4N 3 M5 ON Canada

**Keywords:** Fertility-sparing, Cervical cancer, Trachelectomy, Non-radical, Neoadjuvant chemotherapy, Assisted reproductive technologies, Quality of life

## Abstract

**Background:**

Cervical cancer is the fourth most common cancer among women worldwide, many of who are still within their reproductive lifespan. Advances in screening and treatment have increased the 5-year survival for early stage disease to over 90 % in developed countries. The focus is now shifting to reducing morbidity and improving fertility outcomes for cervical cancer patients. Radical trachelectomy with lymph node assessment became the standard of care for selected women with lesions <2 cm who desire fertility preservation. However, several questions still remain regarding the degree of surgical radicality required for tumors <2 cm, and fertility-sparing options for women with early-stage disesase ≥2 cm, and those with more advanced disease. Here, we compile a narrative review of the evidence for oncologic and pregnancy outcomes following radical trachelectomy, non-radical fertility-sparing surgery, and the use of neoadjuvant chemotherapy prior to surgery for larger lesions. We also review the literature for assisted reproductive technologies in women with more advanced disease.

**Findings:**

Available literature suggests that the crude recurrence and mortality rates after radical trachelectomy are <5 and <2 %, respectively (approx. 11 and 4 % for tumors ≥ 2 cm). Among 1238 patients who underwent fertility-sparing surgery for early cervical cancer there were 469 pregnancies with a 67 % live birth rate. Among 134 cases with lesions ≥ 2 cm, there were ten conceptions with a live birth rate of 70 %. Outcomes after non-radical surgery (simple trachelectomy or cervical conization) are similar, although only applicable among a highly selected patient population. For patients ineligible for fertility-preserving surgery or who require adjuvant radiation therapy, current options include ovarian transposition and cryopreservation of oocytes or embryos but other techniques are under investigation.

**Conclusion:**

Today, many cervical cancer survivors have successful pregnancies. For those with early-stage disease, minimally invasive and fertility sparing techniques have resulted in improved obstetrical outcomes without compromising oncologic safety. Results from three ongoing trials on non-radical surgery for low-risk tumors <2 cm will further inform the need for radical surgery in such patients. For those in whom natural childbearing is unachievable, advances in assisted reproductive technologies provide reproductive options. Despite our advances, the effects of cervical cancer survivorship on quality of life are not fully elucidated.

## Background

Cervical cancer is the fourth most common cancer in women worldwide, with over half a million new cases diagnosed annually [1]. It affects women at a significantly younger age than most other malignancies. According to the Surveillance, Epidemiology, and End Results (SEER) database, between 2008 and 2012, 39 % of new cases diagnosed in the US were in women under the age of 45 [2]. Over the past several decades, most developed countries have seen a significant reduction in overall mortality with 5-year survival rates for localized disease surpassing 90 % [2]. Combined with a trend towards delayed childbearing, this has resulted in a cohort of cervical cancer survivors who are still well within their reproductive lifespan.

Loss of fertility, regardless of cause, is a source of significant psychological distress among women [3]. Several studies suggest that cervical cancer survivors have significantly more reproductive concerns, compared to age-matched controls, including grief about inability to bear children, and an inability to talk openly about fertility [3, 4]. In 2006 the American Society of Clinical Oncology highlighted the importance of addressing future fertility and potential fertility preservation options with patients prior to cancer therapy [5].

Radical trachelectomy (RT) was first described by Eugen Aburel for the treatment of early-stage cervical cancers in the 1950s [6]. This technique was all but forgotten until the 1990s when it was revitalized by Dargent et al. in 1994 [7], to preserve fertility in selected cases through a vaginal approach (VRT). This ushered in a new era of fertility-sparing options for women with early-stage cervical cancer. Over two decades of accumulated data show that for women with small volume disease, this procedure has acceptable surgical morbidity and oncological outcomes [8]. As a result, radical trachelectomy, with pelvic lymph node assessment, became the standard of care for selected women early-stage with disease <2 cm who desire to maintain their fertility [9].

However, several questions still remain about the degree of radicality required, as well as the optimal management of lesions greater than 2 cm. There continues to be a push towards less invasive procedures to reduce peri-operative morbidity, and reduce preterm delivery and perinatal morbidity without compromising oncologic safety. The purpose of this review is to examine the current state of fertility sparing management of cervical cancer, including management of ≥2 cm early stage disease and novel technologies in assisted reproduction for women with locally advanced disease.

## Main text

### Methods

We searched Ovid EMBASE (from 1974 to 2016 week 13) and Ovid MEDLINE in process & other non-indexed citations (from inception to March 2016) for relevant citations. We performed key-word searches combining various disease-specific terms (e.g. cervical cancer, uterine cervix carcinoma) with treatment specific terms (e.g. trachelectomy, conization, neoadjuvant chemotherapy). We limited our search to English language studies. To identify ongoing planned or unpublished trials, we searched the US National Institute of Health’s clinical trial registry at ClinicalTrials.gov. All searches were supplemented by hand searching the reference lists of key papers for relevant citations. Articles were organized based on topics deemed to be relevant by the authors. Crude recurrence, mortality, and birth rates were calculated from large reports of radical trachelectomy overall, radical trachelectomy for lesions ≥2 cm, non-radical fertility sparing procedures, and fertility-sparing procedures after neoadjuvant chemotherapy. Where follow-up studies were available, we included only the most recent and complete series to avoid double counting patients. A narrative review of these topics is presented here.

### Early-stage disease

Early-stage cervical cancer includes disease that is confined to the cervix, measuring ≤ 4 cm, with no apparent spread to adjacent structures or distant organs (International Federation of Gynecology and Obstetrics- FIGO- stages IA1-IB1) [10]. Given the low risk (≤1 %) of either pelvic lymph node or parametrial involvement in stage 1A1 squamous cell carcinoma of the cervix, standard treatment usually consists of cone biopsy or extrafascial hysterectomy, depending on the patient’s desire for fertility preservation [10, 11]. Traditional thinking has dictated that beyond stage IA1 (or in the presence of other high risk features) the increased risk of local spread to the parametria and upper vagina necessitates a more radical surgical approach including lymph node assessment.

Excellent oncological outcomes have been obtained with radical hysterectomy accompanied by bilateral pelvic lymph node dissection for early-stage disease. Five-year overall survival rates range from 73 to 98 % [12–14]. However, this procedure carries a significant risk of surgical morbidity, including increased blood loss, transfusion, and injuries to the bladder, bowel, ureters, and obturator nerve [15–17]. Long-term bladder, anorectal, and sexual dysfunction have been described [18–20]. Over time, minimally invasive and nerve sparing approaches have been developed to reduce morbidity [10], and the degree of surgical radicality required has been challenged with favourable survival and recurrence rates [21, 22].

### Eligibility for fertility sparing management

To be eligible for fertility sparing management of cervical cancer, two main criteria must be met; 1) the patient’s desire for, and likelihood of, fertility must be sufficiently high, and 2) oncologic safety must be acceptable. A pre-operative fertility workup may be considered, particularly in those with a history of infertility [23]. The patient’s age should be considered with respect to ovarian reserve and the increased risk of pregnancy complications with advanced maternal age [23, 24]. Eligibility for fertility-sparing surgery should reflect local jurisdicational age-related eligibility policies for assisted reproductive technologies.

Oncologic safety can be assessed in terms of clinico-pathologic risk factors for recurrence. These include tumour size, depth of stromal invasion, presence of lymphovascular space invasion (LVSI), lymph node and parametrial involvement and the feasibility of achieving tumour-free margins [25–27].

Clinical stage, including vaginal and parametrial spread, should be assessed by an expert (e.g. Gynecologic Oncologist) [23]. Although tumours > 2 cm are at greater risk of lymph node metastasis and recurrence [28, 29], there is a general consensus that larger, expohytic tumours with minimal stromal invasion may be considered for fertility-sparing procedures [30, 31]. In a small prospective trial of 30 patients desiring fertility-preservation, magnetic resonance imaging was shown to have 100 % positive and negative predictive value in assessing suitability for radical trachelectomy [32].

For early stage disease, the difference in recurrence and mortality between squamous carcinomas and adenocarcinomas seems negligible, and both may be considered as candidates for fertility-preservation [33, 34]. A review at our centre found no significant difference in recurrence free survival between 74 patients with adenocarcinoma and 66 patients with squamous cell carcinoma treated with radical vaginal trachelectomy [35]. Although there have been reports of higher incidence of ovarian involvement in adenocarcinoma of the cervix, compared to squamous carcinomas, the overall risk remains low. A review by Touhami and Plante reported a 2 % incidence of ovarian metastasis among those with stage 1B adenocarcinomas of the cervix, among which 96.7 % had other clinical and pathologic features that would preclude fertility-sparing [36]. The authors argue that the risk of surgical menopause in the pre-menopausal population eligible for fertility-sparing outweighs the risk of ovarian involvement, and therefore advocate that ovarian preservation remains an option in the case of early adenocarcinomas of the cervix.

However, non-squamous, non-adenocarcinomas have significantly worse prognosis [37]. In a few early series that included neuroendocrine carcinomas for fertility-sparing, rapid recurrence was observed [28–30]. Many have thus questioned the inclusion of these aggressive histologies in fertility sparing procedures. LVSI alone should not preclude fertility-sparing management. A review by Beiner et al. found that among patients undergoing radical vaginal trachelectomy, 28 % had LVSI, and only 5 % had nodal metastases [38]. While exclusion on this basis is unmerited, extensive LVSI does put these patients at increased risk of nodal involvement [39].

Little prospective data exists as to the optimal surgical margin for trachelectomy specimen. A retrospective review by McCann et al. found that for patients with stage 1A2–2A cervical cancer undergoing radical hysterectomy, close surgical margins (defined as margins ≤5 mm), while not an independent risk factor for recurrence, were associated with other intermediate and high risk features, including lymph node positivity, parametrial involvement, increased size of primary lesion, increased depth of stromal invasion and LVSI [40]. Coincidently, most experts in the area had previously empirically adopted 5 mm as the minimum margin [41, 42]. Therefore, based on extrapolation of data on recurrence following radical hysterectomy and the above empiricism, we believe that optimal surgical margins after fertility-sparing management are at least 5 mm.

### Lymph node assessment

Assessment for lymph node metastases is critical for any patient with greater than 3 mm depth of invasion (i.e. > stage IA1) or other high risk features (e.g. LVSI, high-risk histologies) on a biopsy specimen. Suspicious nodes should be sought on pre-operative CT or MRI but the sensitivity and specificity of these modalities in early cervical cancer is modest, given the low prevalence of enlarged nodes in this population [11]. Combined PET-CT may identify small metastases, however the utility and significance of these remains controversial, particularly in those receiving neoadjuvant chemotherapy prior to surgical management [43].

In the absence of grossly positive nodes on imaging, definitive nodal assessment must be made operatively. Frozen section may be utilized to assess for nodal metastases and positive surgical margins, in which case fertility-sparing surgery may be aborted, or the surgical procedure altered (eg. complete ipsilateral pelvic and para-aortic lymphadenectomy). However, frozen section is not universally practiced due to the concerns regarding false negatives, and loss of tissue for permanent pathological processing. A range of false negative rates for intra-operative frozen section in early cervical cancer has been reported. For stage 1A2–1B1, Panici et al. report a false negative rate of 4.2 % [44]; for stage 1B1–2B, Scholz et al. report a false negative rate of 19 % [45]. Since 2015, the National Comprehensive Cancer Network (NCCN) recommends the consideration of sentinel lymph node procedure (SLNP) for early-stage cervical cancer measuring less than 2 cm [9]. Gortzak examined the use of sentinel lymph node procedure among 81 women undergoing successful sentinel lymph node procedure for early cervical cancer (stage 1A–1B1). They reported a false negative rate of 21.4 % (3/14 negative sentinel nodes). Two of the three cases involved micrometastases <2 mm found only after ultrastaging, highlighting the importance of this element of the sentinel lymph node procedure [46]. Despite a high false negative rate, intraoperative frozen section remains useful in this setting, due to the low prevalence of nodal involvement in early cervical cancer. In this case, the finding of a negative node on frozen section has a negative predictive value of over 97 % [47, 48].

It is recommended that both intraoperative, and final pathology be reviewed by a pathologist specializing in gynecology and that ultrastaging be performed for sentinel lymph nodes. Ultimately, the clinician needs to evaluate all available information, and come to a decision regarding further surgery to define the extent of disease (staging), versus adjuvant therapy be it chemo, radiation, or both- the former not necessarily precluding fertility sparing.

### Radical fertility-sparing surgical management

Radical vaginal trachelectomy (VRT) accompanied by laparoscopic pelvic lymph node dissection has become an accepted treatment modality for fertility preservation in early cervical cancer measuring <2 cm. In 2007, a review of 520 cases found a recurrence and mortality rate of 4.2 and 2.8 %, respectively [49].

Radical trachelectomy can also be performed abdominally (ART), laparoscopically (LRT), and robotically (RRT). An advantage to these alternative approaches is that they more closely resemble the radical hysterectomy familiar to gynecologic oncologists, and do not require special skills in vaginal surgery [34]. Additionally, an abdominal approach allows for potentially greater parametrial resection compared to the vaginal approach [50]. In 2013, Cao et al. performed a matched case-control study comparing surgical approaches in 126 patients undergoing radical trachelectomy [51]. They found no significant differences between VRT and ART for mean operating time, perioperative complications or postoperative complications. Although VRT resulted in higher pregnancy rates (35.5 v. 8.8 %) and live birth rates (23.3 v. 8.8 %), it also resulted in higher rates of recurrence (9.8 v. 0 %) and death from disease (2.8 v. 0 %) [51]. Our review of large case series’ of radical trachelectomy identified oncologic outcomes in 1312 patients eligible for fertility sparing management of early cervical cancer (Table 1). After accounting for adjuvant treatments, 91 % successfully preserved their fertility. The crude recurrence and mortality rates in this group were 4.5 and 1.7 %, respectively. We identified 13 studies that reported individual-level data on recurrences after radical trachelectomy. Fifty-six patients recurred at a median of 18 months after surgery (range 3–108 months). The majority (66 %) of recurrent cases had evidence of intermediate or high-risk features on surgical pathology, or a non-squamous, non-adenocarcinoma histology (Table 2).

**Table 1:** Oncologic outcomes after radical trachelectomy (where *N* > 100 reported)

Study	Eligible for fertility sparing (N)	Stage (N)	Histology (N)	LVSI+ (N)	Approach	LN+ (N)	Required adjuvant therapy (N)	Successful fertility sparing (N)	Primary recurrences (N) (mos)	Dead of disease (N) (mos)	Median follow up months (range)
Shepherd 2006 [108]	123	IA2 = 2 IB1 = 121	83 SCC 33 AC 3 AS 4 other	39	VRT	7	11	112	5 (15, 19, 21, 31, 84)	4 (26, 26, 32, 32)	45^a^ (1–120)
Marchiole 2007 [109]	118	IA1 = 10 IA2 = 19 IB1 = 83 IIA = 6	90 SCC 25 AC/AS 3 rare	43	LAVRT	5	8	97	7 (7, 11, 18, 19, 20, 21, 93)	5 (21, 24, 26, 27. 41)	95 (31–234)
Plante 2011 [57]	140	IA1 = 7 IA2 = 30 IB1 = 97 IB2 = 3 IIA = 3	78 SCC 52 AC 10 AS	40	VRT	5	15	110	6 (−)	2 (−)	95 (4–225)
Helpman 2011 [35]	140	All IA-IB	74 AC 66 SCC	55	VRT	8	9	140	8 (−)	0	60 (−)
Wethington 2012 [110]	101	IA1 = 3 IA2 = 8 IB1 = 88 IB2 = 1 IIA = 1	40 SCC 6 AS 54 AC 1 clear cell	47	ART	19	20	70	4 (−)	0	32 (1–124)
Cao 2013 [51]	150	18 IA1 19 IA2 113 IB1	135 SCC 15 AC	8	VRT ART	0	0	150	7 (−)	2 (−)	25 (6–91)
Mangler 2014 [111]	320^¥^	IA1 = 46 IA2 = 68 IB2 = 207	220 SCC 97 AC 5 AS	94	VRT	–	–	320	10 (mean 26.1 month, range 3–108)	5 (16, 19, 22, 29, 30)	48 (0–216)
Hauerberg 2015 [112]	120	CIS = 2 IA1 = 7 IA2 = 8 IB1 = 103	82 SCC 36 AC 2 AS	30	VRT	4	12	108	6 (−)	2 (−)	55.7 (5.5–147)
Vieira 2015 [113]	100	IA1 = 6 IA2 = 25 IB1 = 69	49 SCC 42 AC 7 AS 2 mixed	25	ART RRT LRT	2	9	83	0	0	51 (10–147)
Total	*N* = 1312							*N* = 1190	*N* = 53	*N* = 20	
Crude rates (%)									Recurrence rate = 4.5 %^#^	Mortality rate = 1.7 %^#^	

*Abbreviations*: *LVSI+* presence of lymphovascular space invasion, *LN+* lymph node metastasis, *SCC* squamous cell carcinoma, *AC* adenocarcinoma, *AS* adenosquamous carcinoma, *LAVRT* laparoscopic-assisted vaginal radical trachelectomy, *ART* abdominal radical trachelectomy, *VRT* vaginal radical trachelectomy, *RRT* robotic radical trachelectomy

^a^Only mean follow up is reported

^#^Crude recurrence and mortality rates among those who successfully had fertility preservation

^¥^In the original study, the sum of the stages and histologies are 321 and 322, respectively, but the reported *N* is 320

**Table 2:** Histopathologic features among recurrences after radical trachelectomy (*N* = 56)

Intermediate/high risk features	*N*
Histology	
Squamous cell carcinoma Adenocarcinoma Adenosquamous Clear cell Neuroendocrine Glassy cell Not reported	26 18 4 1 2 1 4
Size	
≥ 2 cm	20
Lymphovascular space invasion	
positive	22
Lymph nodes	
positive	8
Margins	
positive	1
No intermediate/high risk features	19

Once tumour-free margins (>5 mm) have been achieved, many surgeons insert a cerclage suture around the lower uterine segment, in anticipation of future pregnancy [34]. In an attempt to prevent isthmic stenosis (which occurs in approximately 15 % of cases [52]) we suture a rubber catheter into the os of the lower uterine segment. In our center, this is removed 3 weeks post-operatively [34]. Alternatively, some advocate for the routine use of a temporary intrauterine device for this purpose [53]. If stenosis is suspected, cervical dilatation can be performed [52]. Our review of obstetrical outcomes among 1238 patients who had undergone successful fertility-sparing management for early cervical cancer identified 469 pregnancies, resulting in a 67 % crude live birth rate (Table 3).

**Table 3:** Obstetrical outcomes after radical trachelectomy (where *N* > 50 reported)

Study	Successful fertility sparing management^#^	Attempted to conceive	Conceptions	T1/T2 losses	Live births (ongoing pregnancy)	Median follow up months for entire series (range)
Bernardini 2003 [114]	80	39	22	4	18	–
Hertel 2006 [29]	106	–	18	3	12 (3)	29 (1–128)
Shepherd 2006 [108]	112	63	55	–	28 (3)	45^a^ (1–120)
Li 2011 [53]	56	10	2	0	1 (1)	23 (1–78)
Plante 2011 [57]	110	–	106	29	77	95 (4–225)
Kim 2012 [115]	77	35	27	7	20	–
Wethington 2012 [110]	70	38	31	9	16 (6)	32 (1–124)
Cao 2013 [51]	150	77	20	9	14	25 (6–91)
Nishio 2013 [116]	114	69	31	5	21 (5)	33
Vieira 2015 [113]	83	34	19	5	10 (4)	51 (10–147)
Hauerberg 2015 [112]	108	72	77	21	53 (3)	55.7 (5.5–147)
Kasuga 2016 [117]	172	109	61	13	43 (5)	–
Total	1238	546	469	105	313 (30)	
Crude rates (%)				TI/T2 loss rate = 22.4 %	Live birth rate = 66.7 % &	

*Abbreviations*: *T1* first trimester, *T2* second trimester

^a^Only mean follow up is reported

^#^Excludes those who had completion hysterectomy, or received fertility-compromising adjuvant treatment

& Does not include ongoing pregnancies

Regardless of the approach, higher recurrence rates have been found in patients with larger tumours [51]. Our review of the literature identified 189 cases (for which individual-level data was extractable) of lesions >2 cm eligible for radical trachelectomy (Table 4). Among those who successfully underwent fertility sparing surgery, we identified an overall crude recurrence rate of 11 % and a crude disease-related mortality rate of 4 %. Many feel that lesions ≥2 cm should be triaged to the abdominal approach, where a wider parametrial resection is more attainable [50, 54]. The use of neoadjuvant chemotherapy in this population is discussed below. Furthermore, we identified 134 cases of lesions ≥2 cm where fertility sparing management was successful, resulting in ten conceptions, with a live birth rate of 70 % (Table 5). Ultimately, approximately 25–30 % of women who try to conceive post radical trachelectomy will be infertile [52]. Although three quarters of cases can be attributed to cervical factor, the remaining cases are due to other causes, highlighting the importance of a pre-operative fertility workup in some cases [52].

**Table 4:** Oncologic outcomes of radical trachelectomy for lesions ≥ 2 cm (where *N* > 10 reported)

Study	N with lesions ≥ 2 cm who underwent fertility sparing surgery	Approach	N recurrences (mos)	N dead of disease (mos)	Median follow up months for entire series (range)
Marchiole 2007 [109]	21	LAVRT	6 (7, 11, 18, 20, 21, 93)	4 (21, 24, 27, 41)	95 (31–234)
Nishio 2009 [118]	13	ART	5 (4, 8, 14, 18, 23)	0	27 (1–67)
Cao 2013 [51]	48	VRT ART	5 (−)	2 (−)	34.3^a^
Li 2013 [119]	61	ART	0	0	30 (2–108)
Lintner 2013 [120]	31	ART	4 (5, 6, 10, 14)	2 (16, 22)	90 (60–148)
Wethington 2013 [121]	15	ART LAVRT VRT	1 (9)	0	44 (1–90)
Total	*N* = 189		*N* = 21	*N* = 8	
Crude rates (%)			Recurrence rate = 11.1 %^#^	Mortality rate = 4.2 %^#^	

*Abbreviations*: *LAVRT* laparoscopic-assisted vaginal radical trachelectomy, *ART* abdominal radical trachelectomy, *VRT* vaginal radical trachelectomy

^a^Only mean follow up is reported

^#^Crude recurrence and mortality rates among those who successfully underwent fertility sparing surgery, notwithstanding adjuvant treatment received

**Table 5:** Obstetrical outcomes of radical trachelectomy for lesions ≥ 2 cm (where *N* > 10 reported)

Study	Successful fertility sparing management^#^	Attempted to conceive	Conceptions	T1/T2 loss	Live births (ongoing)	Median follow up months for entire series (range)
Cao 2013 [51]	48	24	3	0	3	34.3^a^
Li 2013 [119]	55	9	3	2	1	30.2 (2–108)
Lintner 2013 [120]	31	8	4	1	3	90 (60–148)
Total	134	41	10	3	7	
Crude rates (%)				T1/T2 loss rate = 30 %	Live birth rate = 70 %	

*Abbreviations*: *T1* first trimester, *T2* second trimester

^a^Only mean follow up is reported

^#^Excludes those who had completion hysterectomy, or received fertility-compromising adjuvant treatment

### Non-radical surgical management

Parametrectomy is responsible for the majority of complications related to radical surgery [55]. Among a subgroup of patients with low-risk pathologic features (lesion <2 cm, depth of invasion <10 mm, and negative pelvic nodes), the risk of paramentrial involvement is estimated to be as low as 0.6 % (90 % CI 0–1.1 %) [56]. Furthermore, after diagnostic LEEP/conization procedures, approximately 65 % of radical trachelectomy specimens have no residual disease [57–59]. Therefore, many patients with early cancers are over-treated at the risk of increased surgical morbidity without the benefit of improved oncologic outcomes. A review by Reade et al. identified 341 patients who had undergone simple hysterectomy or simple trachelectomy for the treatment of stage ≥ IA2 cervical cancer. They found a crude recurrence rate of 6.3 %, and a crude disease-related mortality rate of 1.5 % [11], which are comparable to those achieved by radical trachelectomy [49, 51]. Given these findings, non-radical surgery (simple trachelectomy or conization) could be considered for fertility-sparing in the management of small lesions with favourable prognostic features [55, 56, 60].

Our review of the literature identified 203 cases of early-stage cervical cancer eligible for non-radical, fertility-sparing surgery (Table 6). All patients had lesions <2 cm. Sixty patients underwent simple trachelectomy, and 138 underwent conization. Among 185 cases where fertility-sparing was successful, the crude recurrence rate was 2.7 % and the crude mortality rate was 0.5 %. Among 124 women where fertility preservation was successful, we identified 71 pregnancies with a live birth rate of 68 % (Table 7). Both oncologic outcomes and pregnancy rates compare favourably to literature reports of those undergoing radical trachelectomy. However, it should be noted that the available data is from non-randomized studies of highly selected patient populations with more favourable prognostic factors compared to those undergoing radical surgery.

**Table 6:** Oncologic outcomes of non-radical fertility sparing procedures (where *N* > 10 reported)

Study	N eligible	Surgical procedure (includes pelvic LN assessment)	Successful fertility sparing surgery	N recurrences (mos)	N dead of disease (mos)	Median follow up months for entire series (range)
Bisseling 2007 [122]	18	18 cone	18	0	0	72^a^
Rob 2007 [123]	26	15 ST 7 cone	20	1 (14)	0	49 (18–84)
Landoni 2007 [124]	11	11 cone	11	0	0	20 (7–29)
Fagotti 2011 [125]	17	17 cone	13	0	0	16 (8–101)
Maneo 2011 [126]	36	36 cone	31	3 (20, 34, 36)	1 (72)	66 (18–168)
Raju 2012 [127]	15	15 ST	15	0	0	96 (12–120)
Palaia 2012 [128]	14	14 ST	14	0	0	38 (18–96)
Plante 2013 [129]	16	16 ST	16	0	0	27 (1–65)
Andikyan 2014 [130]	10	9 cone 1 cx bx	9	0	0	17 (1–83)
Bouchard-Fortier 2014 [55]	29	29 cone	29	0	0	21 (1–112)
Salihi 2015 [131]	11	11 cone	9	1 (40)	0	58 (13–122)
Total	203	138 Cone 60 ST	185	*N* = 5	*N* = 1	
Crude rates (%)				Recurrence rate = 2.7 %^#^	Mortality rate = 0.5 %^#^	

*Abbreviations*: *ST* simple trachelectomy, *cx bx* cervical biopsy

^a^Only mean follow up is reported

^#^Crude recurrence and mortality rates among those who successfully underwent fertility sparing surgery, notwithstanding adjuvant treatment received

**Table 7:** Obstetrical outcomes of non-radical fertility sparing procedures (where *N* > 10 reported)

Study	Successful fertility sparing management^#^	Conceptions	T1/T2 losses	Live births (ongoing)	Median follow up months for entire series (range)
Bisseling 2007 [122]	18	18	5	13	72^a^
Rob 2007 [123]	20	15	6	8 (1)	49 (18–84)
Landoni 2007 [124]	11	3	0	3	20 (7–29)
Fagotti 2011 [125]	13	2	0	2	16 (8–101)
Maneo 2011 [126]	31	21	6	14 (1)	66 (18–168)
Raju 2012 [127]	15	4	0	4	96 (12–120)
Plante 2013 [129]	16	8	0	4 (4)	27 (1–65)
Total	124	71	17	48 (6)	
Crude rates (%)			T1/T2 loss rate = 23.9 %	Live birth rate = 67.6 % &	

*Abbreviations*: *T1* first trimester, *T2* second trimester

^a^Only mean follow up is reported

^#^Excludes those who had completion hysterectomy, or received fertility-compromising adjuvant treatment

& Does not include ongoing pregnancies

We identified three ongoing prospective trials designed to assess the efficacy of non-radical surgery in the treatment of low-risk early-stage cervical cancer. The SHAPE trial (NCT01658930) is a randomized trial comparing simple hysterectomy to radical hysterectomy (or cone biopsy to radical trachelectomy) in addition to pelvic lymph node assessment for cases of early-stage (IA2–IB1 < 2 cm), low-risk (stromal invasion <10 mm on LEEP/cone, or <50 % on MRI) cervical cancer [61]. The goal is to demonstrate that in selected cases, non-radical surgery (simple hysterectomy or cone biopsy) is non-inferior to the gold standard radical surgery (radical hysterectomy or radical trachelectomy) with respect to oncologic safety. Treatment-related morbidity, quality of life, and cost-effectiveness are also being evaluated. ConCerv (NCT01048853) is a prospective, international, multicenter cohort study. The goal is to assess the oncologic safety and feasibility of simple hysterectomy or cone biopsy for early-stage (IA2–IB1 < 2 cm) low-risk (negative LVSI, negative margins on cone specimen) cervical cancer [62]. GOG 278 (NCT01649089) is a large prospective cohort study [63]. This study’s primary objectives are to examine the changes before and after non-radical surgical treatment (simple hysterectomy or cone biopsy for fertility preservation plus pelvic lymphadenectomy) on functional outcomes of bladder, bowel and sexual function for early stage cervical cancer. Women with stage IA1 (LVSI+) and IB1 (<2 cm) carcinoma of the cervix with ≤ 10 mm of invasion on diagnostic pathology are eligible for entry. After a pre-operative survey on quality of life, women are stratified based on desire to preserve fertility. Those desiring fertility preservation undergo conization, whereas those not desiring future fertility undergo extrafascial hysterectomy. All patients undergo pelvic lymphadenectomy. Patients with high-risk features on final pathology are offered appropriate adjuvant treatment, and are followed for survival only. Otherwise, patients undergoing non-radical (simple hysterectomy) and fertility-sparing (conization) surgery are assessed at routine post-operative visit and every 6 months there-after for validated quality of life measures, including surgical morbidity, sexual function, fertility intentions, reproductive concerns and impact of therapeutic choice overall. Efficacy (recurrence) is an important secondary objective.

It is hoped that these trials will help to define a select group of patients for whom non-radical surgical management is oncologically safe.

### Bulky (2–4 cm) early-stage disease and the use of neoadjuvant chemotherapy

For 2–4 cm FIGO stage IB1 and IIA disease, neoadjuvant chemotherapy (NACT) has been shown to reduce nodal metastases, parametrial infiltration, and overall tumour size, theoretically making otherwise unresectable (for fertility preserving purposes) disease amenable to surgical management [64–66]. Although meta-analysis of the available data has yet to show a survival advantage for the use of NACT in early cervical cancer, its use in the context of fertility preservation has been gaining attention [66].

Our literature review identified 80 cases of ≥2 cm stage IB1–IIA disease eligible for NACT prior to fertility-sparing surgery (Table 8). The crude recurrence rate is 6.3 % and one patient died from her recurrent disease. The use of NACT has resulted in at least 36 pregnancies with a 72.2 % live birth rate (Table 9).

**Table 8:** Oncologic outcomes of fertility-sparing surgery after NACT (where *N* > 5 reported)

Study	N who received NACT	Timing of LN assessment (N positive LN)	NACT regimen	Surgical procedure	N recurrence (mos)	N dead of disease (mos)	Median follow up months for entire series (range)
Maneo 2008 [65]	21	After NACT (2)	TIP/TEP x 3	Cone	0^a^	0	69 (10–124)
Robova 2010 [132]	15	After NACT (1)	TI/TAx3	ST	3 (−)	1 (−)	76.5 (17–142)
Marchiole 2011 [64]	7	After NACT (0)	TIP/TEP *x*2-3	VRT	0	0	22 (5–49)
Vercecllino 2012 [133]	6	Before NACT (0)	1-TP 5-TIP	VRT	0	0	30.6 (8–70)
Lanowska 2014 [134]	20	Before NACT (0)	TIP/TP × 2-3	VRT	1 (20)	0	23 (1–88)
Salihi 2015 [131]	11	Before NACT (1)	2 TIP x3 4 ddCP x3 5 wCP x3	Cone	1 (40)	0	58 (13–122)
Total	*N* = 80				*N* = 5	*N* = 1	
Crude rates (%)					Recurrence rate = 6.3 %^#^	Mortality rate = 1.3 %^#^	

*Abbreviations*: *NACT* neoadjuvant chemotherapy, *LN* lymph node, *ST* simple trachelectomy, *VRT* vaginal radical trachelectomy, *TP* cisplatin + paclitaxel, *TI* cisplatin + ifosfamide, *TA* cisplatin + doxorubicin (for adenocarcinoma), *TIP* cisplatin + paclitaxel + ifosfamide, *TEP* cisplatin + paclitaxel + epirubicin (for adenocarcinoma), *ddCP* dose dense carboplatin + paclitaxel, *wCP* weekly carboplatin + paclitaxel

^#^Crude recurrence and mortality rates among those who successfully underwent fertility sparing surgery, notwithstanding adjuvant treatment received

^a^ *N* = 3 patients developed CIN in the residual cervix

**Table 9:** Obstetrical outcomes of fertility-sparing surgery after NACT (where *N* > 5 reported)

Study	Successful fertility sparing management^#^	Conceptions	T1/T2 losses	Live births (ongoing)	Median follow up months for entire series (range)
Maneo 2008 [65]	16	10	1	9	69 (10–124)
Robova 2010 [132]	12	7	0	6 (1)	76.5 (17–142)
Marchiole 2011 [64]	7	1	0	0 (1)	22 (5–49)
Lanowska 2014 [134]	18	7	2	4 (1)	23 (1–88)
Salihi 2015 [131]	9	11	4	7	58 (13–122)
Total	62	36	7	26 (3)	
Crude rates (%)			19.4 % T1/T2 loss rate	72.2 % live birth rate	

*Abbreviations*: *T1* first trimester, *T2* second trimester

^#^Excludes those who had completion hysterectomy, or received fertility-compromising adjuvant treatment

& Does not include ongoing pregnancies

The timing of nodal assessment with respect to NACT is not standardized. A study by Vercillino et al. in 2012 showed higher rates of recurrence among a subset of women with positive nodes, in whom fertility-sparing surgery was aborted, compared to women with negative nodes that went on to have NACT and VRT. They suggested that nodal assessment prior to NACT identifies a high-risk group in whom fertility preservation should be avoided [43]. Conversely, some contend that the use of NACT prior to lymph node assessment in these patients may result in fewer nodal metastases, and thus a higher number of patients eligible for fertility-sparing surgery [43]. Our review of the literature identified only 3 series where nodal assessment was carried out prior to NACT (Table 6). While nodal involvement is one of the most significant negative prognostic factors in early-stage cervical cancer, up-front lymph node assessment could theoretically be used to tailor NACT regimen, rather than to exclude potential candidates for fertility-sparing surgery [43].

### Advanced-stage disease

For patients who require hysterectomy and/or pelvic radiotherapy, fertility preservation depends on assisted reproductive technologies. Recognized options include oocyte or embryo cryopreservation prior to cancer therapy and ovarian transposition [5, 67]. The American Society for Reproductive Medicine in 2013 argues that given similar fertilization and pregnancy rates to IVF/ICSI with fresh oocytes, oocyte cryopreservation should no longer be considered experimental [68]. Fertility preservation options that are still considered investigations include ovarian tissue cryopreservation/transplantation and uterine transplantation [5, 69, 70].

The estimated lethal radiation dose to destroy 50 % of oocytes is ≤2 Gy [71] and dependent on age, doses as low as 6 Gy can render a woman menopausal, [72, 73]. The uterus also undergoes irreversible damage after doses from 14 to 30 Gy via reduced uterine volume, reduced elasticity of the uterine musculature and uterine vascular damage [71, 74].

Ovarian transposition was developed as a method to protect the ovaries from the effects of radiation. Due to the theoretical risk of remigration of the ovaries, the ASCO recommendations on fertility preservation suggest performing the transposition as close to the radiation treatment date as possible [5]. Even after the ovaries are transposed, short-term hormonal function is preserved in only approximately 50–93 %, with failure likely related to radiation scatter, remigration, and compromised ovarian blood supply [5, 75–80]. For all of the above reasons, we feel it is important that Gynecologic Oncologists familiar with the radiation borders perform such surgeries. Hwang et. al demonstrated that transposing the ovaries more than 1.5 cm above the iliac crest was significantly associated with successful preservation of ovarian function after treatment [81]. Despite increased success with higher fixation, the Royal College of Obstetricians and Gynecologists recommends that oocyte retrieval be considered for cervical cancer patients prior to the administration of radiation therapy due to the significant risk of ovarian failure after transposition [82]. New random-start ovarian hyperstimulation protocols decrease total time for the IVF cycle without compromising oocyte yield and maturity [83]. Apart from the risk of failure of ovarian transposition, there is also a concern about the risk of metastases in transposed ovaries [84–86]. Given that oophorectomy is not part of the standard treatment of cervical cancer, this risk is not considered prohibitive. Regardless, it seems that ovarian transposition is provided to only a small fraction of eligible patients [87]. A study Salih et. al. in 2015 also suggested that few cervical cancer patients who undergo ovarian transposition end up pursuing in vitro fertilization [88]. The reality is that patients who undergo ovarian transposition require a gestational carrier, which is fraught with ethical, financial and legal issues [89]. Although successful pregnancies have been reported for cervical cancer patients after oocyte retrieval from transposed ovaries and transfer to a gestational carrier, these reports are rare [90–92]. It seems that for most women, the main benefit of ovarian transposition is maintenance of hormonal function rather than preservation of fertility.

### Experimental technique for fertility preservation

Ovarian tissue cryopreservation is an experimental technique for fertility preservation. It is sometimes offered to patients who require immediate gonadotoxic treatment of malignancies where there is insufficient time to offer ovulation induction and cryopreservation of oocytes or embryos. Ovarian tissue can be cryopreserved as cortical biopsies, cortical strips or as whole ovaries, but only cortical biopsies and strips have been successfully transplanted after cryopreservation [69]. The ovarian tissue can be transplanted into a pelvic (orthotopic) or extrapelvic (heterotopic) site. When cortical strips are transplanted orthotopically, they are transplanted either into the medulary portion of a remaining ovary or onto the peritoneum of the ovarian fossa [69]. Studies have reported normal menstrual cycles within 4–9 months after transplantation and graft survival from several months up to 7 years [69, 93–96]. There have been at least 24 births reported after orthotopic transplantation of cortical ovarian tissue, but many of these reports are confounded by the presence of native ovarian tissue [69, 95, 97–99]. Locations of heterotopic transplantation of ovarian tissue resulting in restoration of ovarian function include the forearm, abdominal wall and chest wall [69, 100–102]. Although oocyte retrieval and fertilization with IVF have been reported, there have been no reported live births with this technique. The risk of malignancy from autotransplanted ovarian tissue in cancer patients is not clear, but in a systematic review of 289 patients, metastases were common in patients with leukemia, but less common in most other cancers including cervical cancer [103].

Another experimental procedure that has had recent media attention is uterine transplantation. A 1-year follow-up report of the first uterine transplant trial was published in 2015 [104]. The trial involved uterine transplantation in 9 patients with normal ovarian function who had previously undergone IVF and had cryopreserved embryos. One patient had a history of hysterectomy for cervical cancer. Of the 9 transplanted uteri, 2 were removed within the first 6 months, one due to chronic infection and the other due to bilateral uterine vessel thrombosis. In addition to these grade III surgical complications in the recipients, one of the donors developed a ureterovaginal fistula. Participants were placed on an immunosuppressive protocol. After 1 year of follow-up, 5 of the 7 recipients who kept their transplanted uterus experienced rejection episodes which were all asymptomatic and managed with an intensification of the immunosuppressive regimen. Spontaneous menses occurred in all 7 women within 2 months of transplantation. The plan was to transfer embryos after 12–18 months post-transplant, and a 4–6 month rejection-free period, and to remove the uterus after 1–2 successful pregnancies. As of December, 2015 there were apparently 4 healthy babies born to this cohort [70].

### Patient reported outcomes after fertility-sparing management

While we have made impressive technological advances in early cervical cancer management, the long-term effects of cancer survivorship on quality of life are still not fully understood [105]. Several prospective observational studies have assessed patient reported outcomes after fertility-sparing surgery in early cervical cancer. In 2010, Carter et al. conducted a 2-year prospective study assessing the emotional, sexual, and quality of life concerns of women undergoing radical trachelectomy versus radical hysterectomy for treatment of early-stage cervical cancer [105]. Pre-operatively, both groups reported increased depression, distress, and sexual dysfunction. Although these measurements improved over time, they did not differ significantly by surgery type. This highlights the challenges faced by young cancer survivors in general. After a few years of procedural experience with non-radical fertility-sparing surgery, Song et al., in 2013, examined the effects of surgical radicality on sexual functioning [106]. Women who had undergone non-radical surgery experienced less sexual dysfunction than those who had undergone radical surgery. There was no difference between those who had undergone radical trachelectomy versus radical hysterectomy [106]. While these non-randomized studies generate important hypotheses, they are limited by small sample sizes.

In addition to GOG 278, a prospective questionnaire-based study is currently assessing quality of life and sexual function in women who have undergone radical abdominal trachelectomy [107]. It is expected that these studies will shed light on the patient experience, both in terms of fertility-sparing and non-radical management of early-stage cervical cancer.

## Conclusions

Today, many cervical cancer survivors have the option of becoming a parent. For those with early-stage disease, minimally invasive and fertility sparing techniques have resulted in improved obstetrical outcomes without compromising oncologic safety. For others, natural fertility and childbearing may be unachievable. However, advances in assisted reproductive technologies continue to make pregnancy and/or parenthood a possibility for those who desire it.

Several questions still remain. The safety of non-radical, fertility-sparing surgery has mainly been demonstrated in the context of small, non-randomized comparisons that are fraught with selection bias. The appropriate timing of NACT with respect to nodal assessment, in this context, has yet to be elucidated. The significance of nodal micrometastases remains unclear. For those fortunate enough to undergo fertility preservation and achieve pregnancy, management should be standardized. Centres of excellence should be established, involving gynecologic oncologists, reproductive endocrinologists, maternal fetal medicine specialists, and psychologists specializing in sexual and reproductive health. Ultimately, as we continue to seek answers to our objective questions, the patient-centered purpose of this quest should not be forgotten.

## Abbreviations

AC: Adenocarcinoma
ART: Abdominal radical trachelectomy
AS: Adenosquamous carcinoma
ASCO: American Society of Clinical Oncology
CT: Computed tomography
Cx bx: Cervical biopsy
FIGO: International Federation of Gynecology and Obstetrics
GOG: Gynecologic Oncology Group
Gy: Gray
ICSI: Intracytoplasmic sperm injection
IVF: In vitro fertilization
LARVT: Laparoscopic-assisted vaginal radical trachelectomy
LEEP: Loop electrical excision procedure
LN: Lymph node
LRT: Laparoscopic radical trachelectomy
LVSI: Lymphovascular space invasion
MRI: Magnetic resonance imaging
NACT: Neoadjuvant chemotherapy
NCCN: National Comprehensive Cancer Network
PET-CT: Positron emission tomography-computed tomography
RRT: Robotic radical trachelectoomy
RT: Radical trachelectomy
SCC: Squamous cell carcinoma
SEER: Surveillance, Epidemiology, and End Results database
SLNP: Sentinel lymph node procedure
ST: Simple trachelectomy
T1: First trimester
T2: Second trimester
VRT: Vaginal radical trachelectomy
